# Minoxidil Topical Foam: A New Kid on the Block

**DOI:** 10.4103/0974-7753.58560

**Published:** 2009

**Authors:** Jaideep A Gogtay, Madhumita Panda

**Affiliations:** Medical Department, Cipla Ltd., Mumbai Central, Mumbai - 400 008, Maharashtra, India

Androgenetic alopecia or pattern hair loss results from miniaturization of hair follicles in androgen-sensitive areas of the scalp in genetically predisposed persons. Arresting the process of miniaturization remains the goal of medical treatment.

Topical minoxidil and oral finasteride are the only options currently approved by the Food and Drug Administration (USA). Following the initial reports in 1980, minoxidil topical solution (MTS) has been a proven mainstay of treatment for androgenetic alopecia.

Adverse effects with MTS are irritation of the scalp, including dryness, scaling, itching and redness, occurring in approximately 7% of patients who use the 2% solution and in more of those who use the 5% solution because of its higher content of propylene glycol.[1] The vehicle in MTS consists of water, alcohol and propylene glycol, of which propylene glycol was found to be the agent most frequently responsible for allergic contact dermatitis.[2]

Minoxidil has now been incorporated in a foam formulation (Minoxidil topical foam, MTF), which is propylene glycol free. It is a liquid pressurized in an aluminum can with a hydrocarbon propellant, which on valve actuation forms a foam lattice. This matrix is thermolabile and breaks down rapidly as soon as it comes in contact with skin. The volatile constituents evaporate and, within 20-30 s, little or no residue remains on the skin. This causes the active ingredient to concentrate at the vehicle-skin interface, and facilitates penetration. Foam has the ability to deliver a greater amount of the active drug at an increased rate compared with other vehicles.[3]

In the hamster ear model, 5% MTF showed increased uptake of minoxidil by five times over 5% MTS at 2 h of application.[4] A human pharmacokinetic study showed that systemic absorption of minoxidil with 5% MTF (1 g twice daily) was about half of that with 5% MTS (1 cc twice daily).[5] Another exaggerated-use pharmacokinetic study in men showed that application of 3 gm of MTF twice daily resulted in systemic levels that were below the 21 μgm/mL threshold for cardiac-related events.[5]

A double-blind, placebo-controlled study in 352 men of 16 weeks duration showed a 13.4% increase in target area hair count with 5% MTF compared to 3.4% with placebo. 70.6% of patients felt that hair loss had improved from baseline.[4] The incidence of adverse events (stinging, burning and itching) was similar in the placebo and 5% MTF group. The incidence of pruritus with 5% MTF was 1.1% vs. 6% seen in a separate trial of 5% MTS.[4] Although there is no comparative clinical study, a study in macaques comparing the two formulations showed an increase in hair weight of 12.4 mg with 5% MTF and 9.27 mg with 5% MTS.[6]

Studies involving patient feedback showed a significant preference to MTF on several aesthetic attributes, including ease of application, lack of dripping, quick drying and ability to fit easily into a daily routine.

This propylene glycol free thermolabile, hydroethanolic formulation of minoxidil offers a new option for patients with androgenetic alopecia.
